# Learning ecosystem-scale dynamics from microbiome data with MDSINE2

**DOI:** 10.1038/s41564-025-02112-6

**Published:** 2025-09-09

**Authors:** Travis E. Gibson, Younhun Kim, Sawal Acharya, David E. Kaplan, Nicholas DiBenedetto, Richard Lavin, Bonnie Berger, Jessica R. Allegretti, Lynn Bry, Georg K. Gerber

**Affiliations:** 1 Division of Computational Pathology, Brigham and Women’s Hospital, Boston, MA USA; 2 Broad Institute of MIT and Harvard, Cambridge, MA USA; 3 Harvard Medical School, Boston, MA USA; 4 Computer Science and Artificial Intelligence Lab, MIT, Cambridge, Boston, MA USA; 5 Mathematics Department, MIT, Cambridge, MA USA; 6 Massachusetts Host-Microbiome Center, Brigham and Women’s Hospital, Boston, MA USA; 7 Harvard-MIT Health Sciences and Technology, Cambridge, MA USA; 8 Division of Gastroenterology, Brigham and Women’s Hospital, Boston, MA USA

**Keywords:** Machine learning, Bayesian inference, Ecological networks, Dynamical systems

## Abstract

Although dynamical systems models are a powerful tool for analysing microbial ecosystems, challenges in learning these models from complex microbiome datasets and interpreting their outputs limit use. We introduce the Microbial Dynamical Systems Inference Engine 2 (MDSINE2), a Bayesian method that learns compact and interpretable ecosystems-scale dynamical systems models from microbiome timeseries data. Microbial dynamics are modelled as stochastic processes driven by interaction modules, or groups of microbes with similar interaction structure and responses to perturbations, and additionally, noise characteristics of data are modelled. Our open-source software package provides multiple tools for interpreting learned models, including phylogeny/taxonomy of modules, and stability, interaction topology and keystoneness. To benchmark MDSINE2, we generated microbiome timeseries data from two murine cohorts that received faecal transplants from human donors and were then subjected to dietary and antibiotic perturbations. MDSINE2 outperforms state-of-the-art methods and identifies interaction modules that provide insights into ecosystems-scale interactions in the gut microbiome.

## Abstract (web summary)

This Bayesian statistical method uses timeseries microbiome data to infer interaction modules and is tested using a faecal transplant experiment in mice.

## Main

Microbiomes are inherently dynamic^1^, changing over time due to both microbial interactions and responses to external perturbations. The dynamics of a microbiome reveals important information not only about the individual microbial constituents, but also about how the ecosystem as a whole behaves; for instance, unstable responses of the ecosystem to perturbations can indicate an inability to maintain homeostatic function^2^. Mathematical models of dynamical systems have a long history in ecology and biomedicine, and have led to many insights, including for microbial ecosystems^3^. Dynamical systems models are time causal, meaning that they predict future observations from past inputs. This type of model is particularly powerful because, once inferred from data, it can be directly interrogated using mathematical tools or computational simulations to study aspects including stability and other ecological properties^4–7^; topological properties of the interaction network such as motifs^8–10^; and in silico forecasts of the system such as ‘knockouts’ of taxa or responses to perturbations not yet experimentally studied. Methods that are not time causal have also been used to analyse longitudinal microbiome data, such as approaches that regress against time^11–13^. Although these approaches are useful for applications such as clustering or de-noising timeseries data, their dependence on future information means that they cannot be used to study the formal ecosystem properties or ‘what-if’ types of analysis described above.

Despite the promise of dynamical systems models, they are challenging to apply to the mammalian microbiome because of the scale and complexity of these ecosystems and limitations of data sources. A well-established dynamical systems modelling framework, the generalized Lotka–Volterra (gLV) equations^14–17^, models pairwise interactions among microbial taxa. Although gLV models have been shown empirically to predict microbial dynamics with good accuracy for small ecosystems^16^, these models present challenges for scalability and interpretability, because the number of modelled interactions increases quadratically with the number of taxa in the system. Measurement noise also presents challenges for inferring models, with sequencing data having complex noise characteristics that can disproportionately affect low abundance but ecologically important taxa^18,19^. In addition, appropriately designed experiments are critical for learning dynamical systems models from microbiome data^20^. When an ecosystem is changing minimally, it effectively only provides information about a single ecological state, for example, when it is at equilibrium. In contrast, when an ecosystem is perturbed from its equilibrium state, its transient behaviour is revealed, which is especially informative about how components of the system interact. A related issue is that longitudinal experiments must be designed to sample at sufficient temporal frequencies to capture transient behaviours, particularly around the times of perturbations^20^.

In this work, we provide two key resources to the community to address the challenge of inferring microbiome dynamical systems from data at ecosystem scale: (1) Microbial Dynamical Systems Inference Engine 2 (MDSINE2), implemented as an open-source software package with extensive analysis and visualization options, providing notable innovations over the state-of-the-art^14–17^, and (2) two new microbiome longitudinal datasets specifically designed for dynamical systems inference, which to our knowledge are the most temporally densely sampled microbiome datasets so far that also include intentionally introduced perturbations. The remainder of this manuscript is organized as follows. First, we describe the MDSINE2 method and new high-temporal-resolution longitudinal microbiome datasets. Next, we benchmark MDSINE2 against state-of-the-art methods on real and simulated data. Finally, we conduct a case-study analysis applying MDSINE2 to a high-temporal-resolution dataset to demonstrate its ability to yield interpretable and biologically relevant findings.

## Results

### Open-source computational tool for learning microbiome dynamics

**Fig. 1:**
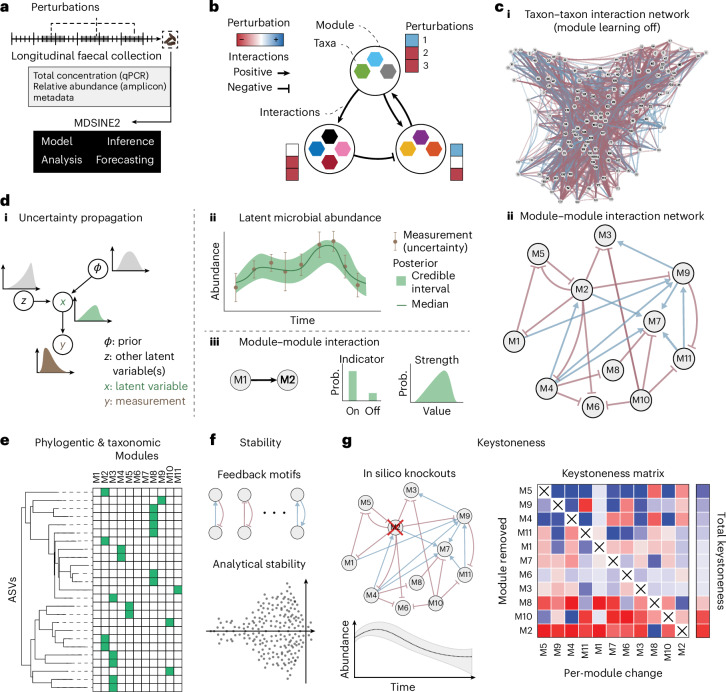
Schematic of the MDSINE2 method for inferring interpretable dynamical systems models of microbiomes at scale. **a**, Input data are measurements of total bacterial concentration (for example, 16S rRNA gene quantitative polymerase chain reaction (qPCR)) and measurements of taxa abundances (for example, 16S rRNA gene amplicon sequencing). Measurements should be obtained from studies in which the microbiome undergoes perturbations, providing effective information for inference. **b**, MDSINE2 infers dynamical systems models from data with the option of automatically learning interaction modules, or groups of taxa that share the same interactions with other modules and perturbations. This is a more compact representation that is more readily interpretable than learning interactions among all microbes. **c**, Example microbial interaction networks for the same number of taxa without module learning (**i**) and with module learning (**ii**). **d**, MDSINE is fully Bayesian and propagates error throughout the model (**i**), providing estimates of uncertainty for all variables (for example, latent trajectory along with measurements and their uncertainty (**ii**), and indicator and interaction strengths for ecological interactions (**iii**)). Prob., probability. **e–g**, The software provides a variety of tools for analysis and visualization of the inferred dynamical system, including analyses of taxonomic composition and phylogeny of modules (**e**), formal analyses of ecosystem stability and interaction motifs (**f**), and keystoneness (quantitative impact on the ecosystem when modules are removed) (**g**).

To facilitate inference of accurate and interpretable large-scale dynamical systems models from microbiome timeseries data, we developed MDSINE2 (Fig. 1). Inputs to the open-source software package are: (1) timeseries measurements of bacterial abundances in the form of counts (for example, 16S rRNA gene amplicon or shotgun metagenomics data), (2) total bacterial concentrations (for example, from 16S rRNA gene qPCR measurements) and (3) associated metadata for the samples. The software also provides a variety of tools for interpreting the model that it learns from data, including plotting trajectories of taxa, analysing topological properties of the interaction network, quantitating the predicted ecological importance of individual taxa or modules (‘keystoneness’) and formally assessing the stability of the microbial ecosystem (Fig. 1e–g).

MDSINE2 uses a probabilistic machine learning model based on generalized Lotka–Volterra (gLV) dynamics, with several key innovations over state-of-the-art methods^14–17^. First, MDSINE2 employs a fully Bayesian probability model that explicitly models the measurement uncertainty associated with microbiome sequencing and bacterial concentrations (Fig. 1d). An advantage of this approach is that MDSINE2 provides quantitative measures of uncertainty (Bayes factors^21^, which are a Bayesian alternative to *P* values) for all model parameters that can be used to interpret the confidence of predictions and prioritize downstream analyses. Second, MDSINE2 includes stochastic effects in dynamics to capture random fluctuations in microbial trajectories that occur due to unmeasured effects on the ecosystem. Third, MDSINE2 extends the gLV model to automatically learn ‘interaction modules’ (Fig. 1b), which we define as groups of taxa that share common interaction structure (that is, are promoted or inhibited by the same taxa outside the module) and have a common response to external perturbations (for example, antibiotics). Interaction modules are motivated by both empirical observations that groups of microbial taxa covary^22,23^ and theoretical ecology concepts such as guilds (groups of taxa that utilize resources in a similar way)^24^. Modular structure (Fig. 1b,c(ii)) reduces the complexity of the system to be analysed, which increases interpretability^25,26^ and also enables scalability by reducing the number of parameters in the model from order quadratic in the number of taxa (that is, all potential pairwise interactions between taxa in the gLV equations) to order quadratic in the number of modules (which scales logarithmically with the number of taxa)^27^. The number of modules is treated probabilistically with full uncertainty quantification and learned from the data, alleviating the need for the user to pre-specify this information. See Methods section ‘MDSINE2 model’ and Supplementary Text 1 for further details on the model. Details on model inference can be found in Methods section ‘Case-study model inference’ and Supplementary Text 2. Formal sensitivity analysis of model hyperparameters can be found in Supplementary Text 3.

### High-temporal-resolution gut microbiome studies with humanized mice

**Fig. 2:**
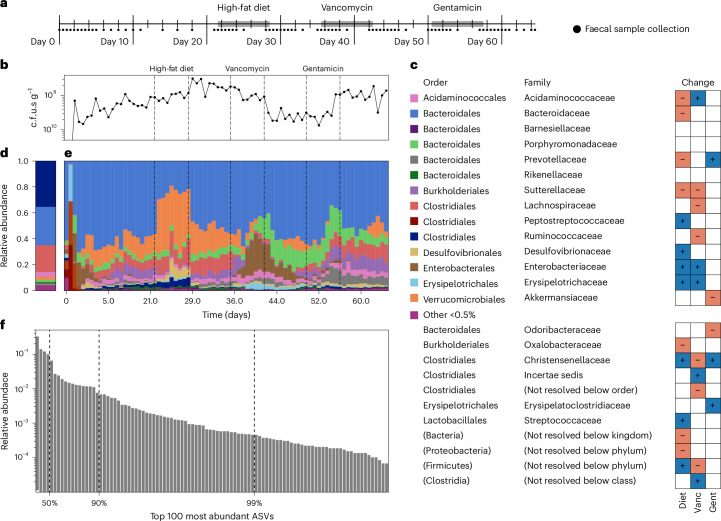
High-temporal-resolution gnotobiotic mice colonization and perturbation study using a healthy human donor microbiome shows reproducible differential responses to perturbations. **a**, Experimental design for the healthy cohort (*n* = 4 mice) with an average of 76 serial faecal samples per mouse. **b**, Average total bacterial concentrations in serial faecal samples. **c**, Legend and taxonomy for **d** and **e** along with significant differential abundances (*P* < 0.05 for two-sided Wald test with BH correction) for each taxonomic group across the three perturbations (see Supplementary Data for *P* values). Vanc, vancomycin; Gent, gentamicin. **d**, Relative abundance of microbes in human donor sample. **e**, Relative abundances of microbes in serial faecal samples, averaged over the biological replicates. **f**, Relative abundances of the top 100 most abundant ASVs across all mouse samples. Source data

**Extended Data Fig. 1:**
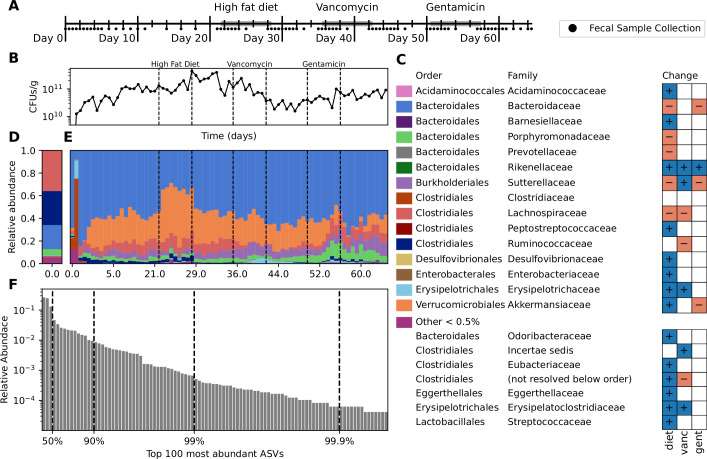
High-temporal resolution gnotobiotic mice colonization and perturbation study using a dysbiotic human donor microbiome shows reproducible differential responses to perturbations. (**a**) Experimental design for the dysbiotic cohort (n = 5 mice) with an average of 76 serial fecal samples/mouse. (**b**) Average total bacterial concentrations in serial fecal samples. (**c**) Legend and taxonomy for panels **d** and **e** along with significant differential abundances (p < 0.05 for two-sided Wald test with BH correction) for each taxonomic group across the three perturbations (see Supplementary Data Table for *p*-values). (**d**) Relative abundance of microbes in human donor sample. (**e**) Relative abundances of microbes in serial fecal samples, averaged over the biological replicates. (**f**) Relative abundances of the top 100 most abundant ASVs across all mouse samples. Source data

**Extended Data Fig. 2:**
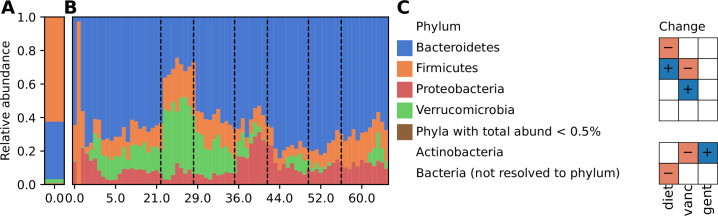
Phylum level grouping of reads for the healthy cohort. (**a**) Relative abundance of microbes in human donor sample. (**b**) Relative abundances of microbes in serial fecal samples, averaged over the biological replicates. **(c)** Legend and taxonomy for panels **a** and **b** along with significant differential abundances for each taxonomic group across the three perturbations (n = 4 mice, p < 0.05 for two-sided Wald test with BH correction). see Supplementary Data Table for *p*-values. Source data

**Extended Data Fig. 3:**
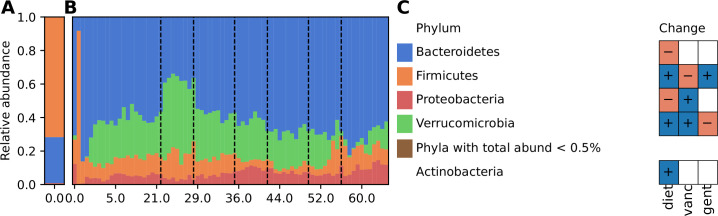
Phylum level grouping of reads for the dysbiotic cohort. (**a**) Relative abundance of microbes in human donor sample. (**b**) Relative abundances of microbes in serial fecal samples, averaged over the biological replicates. (**c**) Legend and taxonomy for panels **a** and **b** along with significant differential abundances for each taxonomic group across the three perturbations (n = 5 mice, p < 0.05 for two-sided Wald test with BH correction). see Supplementary Data Table for *p*-values. Source data

Given the importance of sufficient temporal resolution and perturbations for inferring dynamical systems models from data^20^, we generated two new datasets to serve as benchmarking and analysis resources for the community. The data were generated from two cohorts of ‘humanized’ germ-free mice (Fig. 2 and Extended Data Fig. 1) that underwent faecal microbiota transplantation from a healthy human donor (*n* = 4 mice) and a donor with ulcerative colitis (*n* = 5 mice). After an equilibration period of 3 weeks, mice were subjected to a sequence of three perturbations (high-fat diet (HFD), vancomycin and gentamicin). These perturbations were chosen because they differentially affect components of the microbiome (for example, high-fat/simple-carbohydrate versus complex-carbohydrate utilizers and bacteria susceptible or resistant to different antibiotics). Mice were separately housed, and faecal samples were collected over a 65-day duration, with an average of 76 samples per mouse (Fig. 2a and Extended Data Fig. 1a), resulting in a total of 686 faecal samples. The samples were interrogated for relative abundance via 16S ribosomal RNA (rRNA) amplicon sequencing (Fig. 2d,e and Extended Data Fig. 1d,e) and total bacterial concentration via qPCR using a universal 16S rDNA primer (Fig. 2b and Extended Data Fig. 1b). The resulting ~50 million sequencing reads were bioinformatically processed using DADA2 (ref. ^28^) and filtered to yield high-quality timeseries information for dynamical systems inference tasks^20^ (see Methods for details), resulting in 141 amplicon sequence variants (ASVs) in the healthy cohort and 121 ASVs in the ulcerative colitis/dysbiotic cohort. See Supplementary Text 4.1 for further information about basic taxonomic composition and other standard analyses of these datasets (Fig. 2c,f and Extended Data Figs. 1c,f, 2 and 3). For brevity, and because the healthy cohort harboured more taxa and greater microbial diversity, we focus primarily on examples from this cohort in the main manuscript.

### MDSINE2 outperformed state-of-the-art methods forecasting with real data

We evaluated MDSINE2’s performance against state-of-the-art methods on our high-temporal-resolution datasets, using a standard metric in the field, forecasting of held-out microbial dynamics, which does not require ground-truth information and thus allows for benchmarking on real data. Specifically, we employed a one-subject-hold-out training and testing methodology. All data from one mouse in a cohort were held out while the model was trained on the remaining data from the other mice in that cohort. Then, the model forecasts all taxa trajectories for the entire timeseries (except for the first timepoint) using the measured microbial abundances on the first timepoint in the held-out mouse as the initial condition. We evaluated performance using root-mean-squared error (RMSE) of log abundances over the timeseries, a measure of the difference between the predicted and ground-truth measurement. Our experimental data included measurements of total microbial concentrations, which are formally necessary for inference of standard gLV models, including the state-of-the-art ridge regression (gLV-L2) and elastic-net regression (gLV-net)^14^ methods. To assess the impact of interaction modules on model performance and to more directly compare our method to the state-of-the-art methods, such as gLV-L2 and gLV-net which do not infer modules, we also included MDSINE2 without interaction modules (MDSINE2^−M^) as a comparator method.

**Fig. 3:**
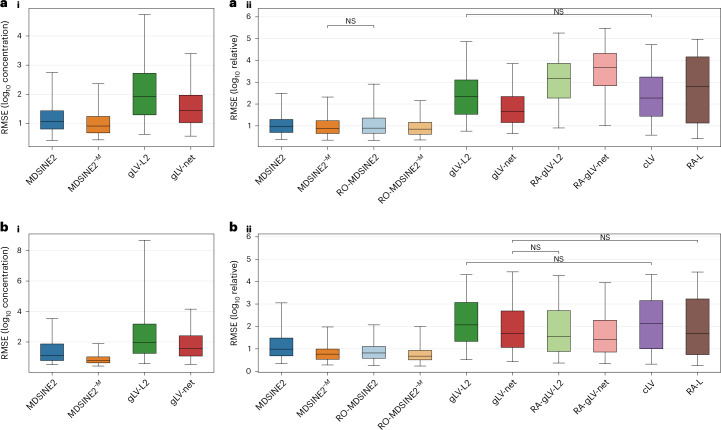
MDSINE2 outperforms state-of-the-art methods in forecasting microbial concentrations and relative abundances. Cross-fold validated RMSE was calculated from forecasted abundances with respect to the held-out mouse (that is, training on all timepoints from the mice not held out, and forecasting on the held-out mouse with only the initial timepoint provided). RMSE are visualized across all folds and for all taxa with non-zero reads within each sample for the (**a**) healthy cohort (*n* = 564, 141 ASVs × 4 mice) and (**b**) dysbiotic cohort (*n* = 605, 121 ASVs × 5 mice). **a**(**i**),**b**(**i**), Models trained on microbial concentrations. **a**(**ii**),**b**(**ii**), Models trained on microbial concentrations or relative abundances/reads-only with the performance metric taken with respect to relative abundances. All pairwise comparisons are significant, with BH-corrected two-sided Wilcoxon signed-rank test *P* < 0.05 unless noted with NS (not significant). Boxes denote interquartile region with a line for the median; whiskers denote 95% interval. All *P* values are provided in Supplementary Data. Source data

MDSINE2 and MDSINE2^−M^ significantly outperformed the two gLV comparator methods that were trained on microbial concentrations for both the healthy and dysbiotic cohorts (Fig. 3a(i),b(i)). MDSINE2^−M^ showed slight but statistically significant, better forecasting accuracy over MDSINE2, consistent with our previous finding that model constraints can impact forecasting performance to some extent^16^. However, it is notable that MDSINE2 uses vastly fewer parameters than the other methods, including MDSINE2^−M^: for the total concentration forecasting task on the healthy cohort, for example, MDSINE2 used only 272 parameters as opposed to 19,740 for MDSINE2^−M^, a >72× reduction. Given that the actual gap in performance between MDSINE2^−M^ and MDSINE was quite minor, our results suggest that the much more compact dynamical system representation learned by MDSINE2 still captures the system behaviour quite accurately. We additionally assessed performance of versions of MDSINE2 and comparator methods on relative abundance (RA) or reads-only (RO) data (that is, not including bacterial concentration information). These results demonstrated that RO versions of MDSINE2 also significantly outperformed all the comparator methods (Fig. 3a(ii),b(ii) and Supplementary Text 4.2).

### MDSINE2 accurately recovers ecosystem dynamics from (semi-)synthetic data

**Extended Data Fig. 4:**
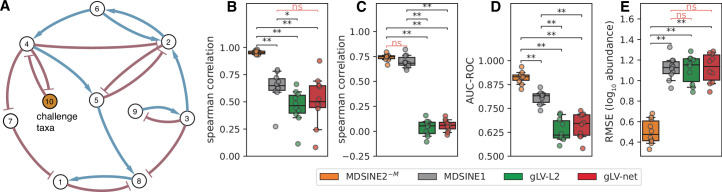
MDSINE2 outperforms state-of-the-art methods on a 10 taxa synthetic benchmarking standard. (**a**) Underlying dynamical systems network topology^16^, which was used to simulate data under a gLV model (n = 10 seeds). Spearman correlation between model predictions for (**b**) growth rate strengths and (**c**) interactions strengths (higher is better, 1 is maximum) (**d**) Area under the receiver operating curve (AUC-ROC) for interaction presence/absence (higher is better, 1 is maximum). (**e**) RMSE of log abundance for model forecast from initial conditions compared to ground truth trajectory (lower is better). Pairwise comparisons performed with BH corrected two-sided Wilcoxon signed-rank test * p < 0.05, ** p < 0.01. Boxes denote interquartile region with a line for the median. Whiskers denote 95% interval. *p* values provided in Supplementary Data Table. Source data

We sought to assess MDSINE’s ability to recover underlying dynamical systems’ parameters and interaction network topologies. This type of analysis requires ground-truth information, which is unavailable for the microbiome. We thus benchmarked our method on fully synthetic and semi-synthetic data. For fully synthetic data, we used a benchmarking standard with 10 taxa that we previously published^16^ and found that MDSINE2 accurately recovered the dynamical system, and moreover significantly outperformed state-of-the-art methods, including our previous MDSINE method, on all metrics (Extended Data Fig. 4).

**Fig. 4:**
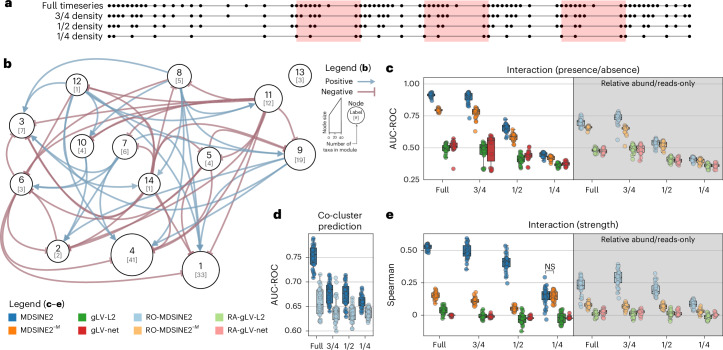
MDSINE2 accurately recovers dynamical systems from semi-synthetic microbiome timeseries data. The parameters of the dynamical systems model inferred by MDSINE2 on the healthy cohort were used to simulate trajectories for the 141 taxa present to create a fully observed dataset. Corrupted versions of these datasets were generated, with experimental noise in both sequencing and qPCR measurements, as well as downsampling of the number of observed timepoints. **a**, Timeseries downsampling scheme. Shaded regions indicate when perturbations were introduced. **b**, The ground-truth network used (nodes are modules, with the number of taxa in each module in square brackets). **c**, AUC-ROC for inferring the presence or absence of taxa interactions. **d**, AUC-ROC for inferring which taxa are in the same module, using taxa co-cluster probabilities. **e**, Spearman rank correlation between ground truth and inferred interaction strengths. MDSINE2 outperformed all other methods in both predicting the presence or absence of an interaction and the strength of those interactions. With module learning off, reducing the number of temporal samples, or without qPCR measurements, MDSINE2’s performance degraded. For all scenarios, *n* = 30 simulations, with 10 different seeds for the initial conditions used to generate trajectories and 3 seeds for measurement simulation (read abundances and qPCR values). Statistical significance tests were performed within each timeseries downsampling scheme for the four methods either trained on all available data or reads/relative abundances. All pairwise comparisons are significant, with BH-corrected two-sided Wilcoxon signed-rank test *P* < 0.05 unless noted with an NS. Boxes denote interquartile region with a line for the median; whiskers denote 95% interval. All *P* values are provided in Supplementary Data. abund, abundance. Source data

**Extended Data Fig. 5:**
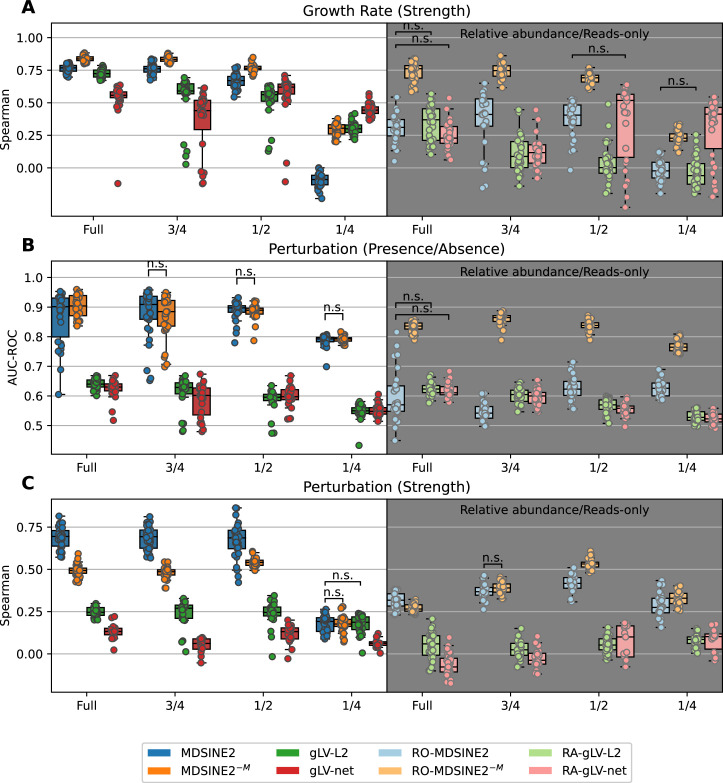
MDSINE2 accurately recovers dynamical ecosystems from semi-synthetic time-series data (growth rates and perturbations). (**a**) Spearman correlation for growth rate strength prediction. (**b**) AUC-ROC for predicting taxa perturbation presence/absence. (**c**) Spearman correlation for predicting taxa perturbation strengths. MDSINE2 and MDSINE2^−M^ outperform all other methods in all scenarios except for the ¼ density temporal sampling regime. For all scenarios n = 30 simulations, 10 different seeds for the initial conditions used to generate trajectories and 3 seeds for measurement simulation (read abundances and qPCR values). Boxes denote interquartile region with a line for the median. Whiskers denote 95% interval. Statistical significance tests were performed within each time-series down sampling scheme for the four methods either trained on all available data or reads/relative abundances. All comparisons are significant (p < 0.05) with BH correction unless denoted with “n.s.”. All *p* values provided in Supplementary Data Table. Source data

For realistically sized microbiome ecosystems, no established benchmarking dataset exists and theoretical principles needed to construct a realistic synthetic microbial ecosystem at this scale remain an active area of research, so we developed a semi-synthetic data generation procedure. Briefly, we used the parameters of the dynamical systems model inferred by MDSINE2 on the healthy cohort as ground-truth information (Fig. 4b) and forward simulated trajectories for the 141 taxa from the model to create a fully observed dataset. We then created corrupted versions of the dataset, with simulated measurement noise added to generate sequencing and qPCR measurements, as well as downsampling of the number of observed timepoints (Fig. 4a, see Methods section ‘Semi-synthetic data and benchmarking’ for complete details). The corrupted datasets were then used to assess the ability of different methods to recover parameters of the underlying dynamical system as shown in Fig. 4 and Extended Data Fig. 5. We assessed MDSINE2’s performance with or without qPCR data as input, as well as performance of the two gLV models and their relative-abundance (RA)-only counterparts. Note that the scale of interactions and perturbation strengths are not identifiable without bacterial concentration measurements. Thus, to enable comparisons between methods on these parameters, we used a popular scale invariant performance metric, the Spearman rank correlation. Higher values of the Spearman rank correlation represent stronger relationships, and a value of zero represents no relationship (random chance). To assess performance on binary inferences (presence/absence of interactions or perturbations and co-clustering of taxa into interaction modules), we used area under the receiver operator characteristic curve (AUC-ROC). An AUC-ROC of 0.5 indicates random chance, with higher AUC-ROC values indicating better performance.

Overall, we found that MDSINE2 accurately recovered key properties of the underlying ground-truth dynamical system (Fig. 4 and Extended Data Fig. 5). On the full timeseries, MDSINE2 accurately recovered microbial interactions both in terms of presence/absence and strength, with a median AUC-ROC of 0.91 and a Spearman rank coefficient of 0.53, respectively (Fig. 4c,e). In addition, MDSINE2 showed strong performance in accurately predicting when two taxa came from the same module (median AUC-ROC of 0.76 on the full timeseries, Fig. 4d). With module learning off, reducing the number of temporal samples, or without qPCR measurements, MDSINE2’s performance degraded. The gLV-L2 and gLV-net methods were essentially unable to recover the interactions under either metric even with the full timeseries available. MDSINE2 and MDSINE2^−M^ additionally significantly outperformed all other methods in recovering growth rates, perturbation strengths and perturbation presence/absence, except for the most sparsely sampled regime with 75% of the timepoints ablated (Extended Data Fig. 5). As with recovering interactions, reducing the number of temporal samples or removing the qPCR measurements typically reduced MDSINE’s performance. We note that while including modules significantly improved MDSINE2’s performance for recovering interactions, this was not always the case for growth rates or perturbations in the scenarios assessed. Overall, these results indicate that MDSINE2 can accurately recover its underlying dynamical systems model from data that has realistic levels of measurement noise and numbers of observed timepoints, and models trained on relative abundances/reads alone significantly underperform compared to models that include bacterial concentration estimates in nearly all scenarios.

### Case study with MDSINE2 on the healthy microbiome cohort

**Fig. 5:**
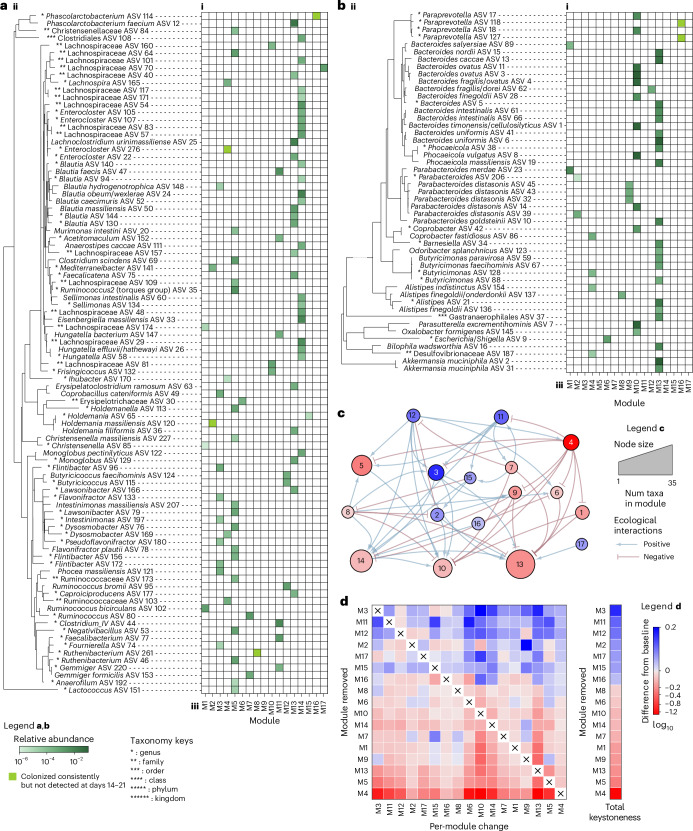
MDSINE2 infers modular representations of complex microbiomial dynamical systems. Our method automatically learns modules of taxa (ASVs) on the basis of similarity of their dynamic interactions and responses to perturbations. Results are split into **a**, Gram-positive, and **b**, Gram-negative ASVs, for display purposes. **a**(**i**),**b**(**i**), ASV relative abundance. **a**(**ii**),**b**(**ii**), Phylogenetic tree of ASVs. **a**(**iii**),**b**(**iii**), Module memberships. Intensity of colour in the grid indicates abundance post colonization and before perturbations (average over days 14–21). **c**, Inferred module interaction network displaying only edges with BF > 100 (‘decisive’ evidence). Size of nodes is proportional to the number of ASVs in the module, and colour of nodes corresponds to module keystoneness. Num, number. **d**, Keystoneness analysis measures the relative importance of a module in maintaining the steady-state abundance of the community. A positive keystoneness for a module means that its removal from the community reduces the steady-state abundance of its community members, and a negative keystoneness means that the module’s removal results in an increase in the abundance of the other community members. A module’s impact on the other community members was computed both at the module level and across all modules (total keystoneness). Source data

**Extended Data Fig. 6:**
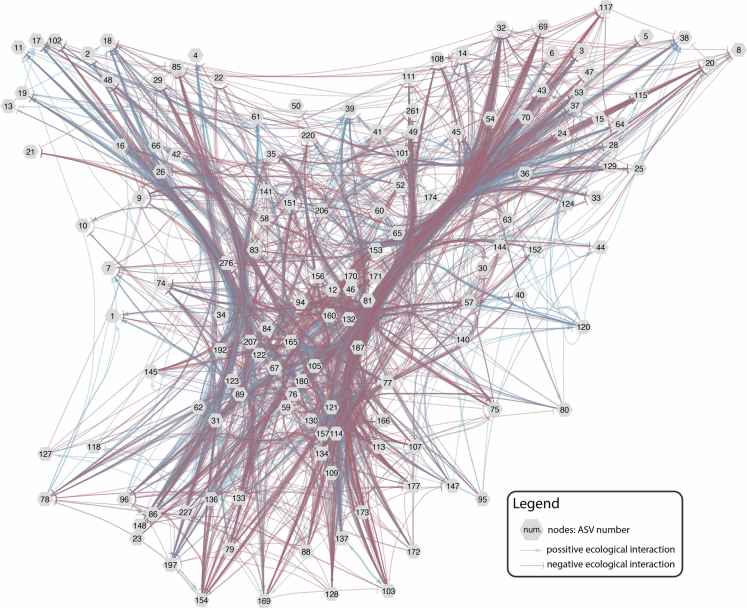
Taxon-taxon interaction network for model trained without module inference on healthy cohort. Interaction network displaying only edges with BF > 100 (*decisive* evidence).

**Extended Data Fig. 7:**
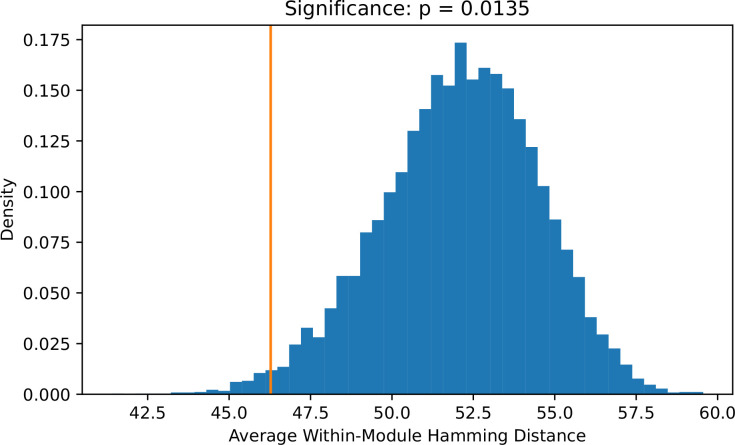
Phylogenetic distance of modules. Average phylogenetic distance of modules shown by orange line, compared to random permutations of taxa in modules for null distribution in blue. Permutation test results in p = 0.0135.

**Extended Data Fig. 8:**
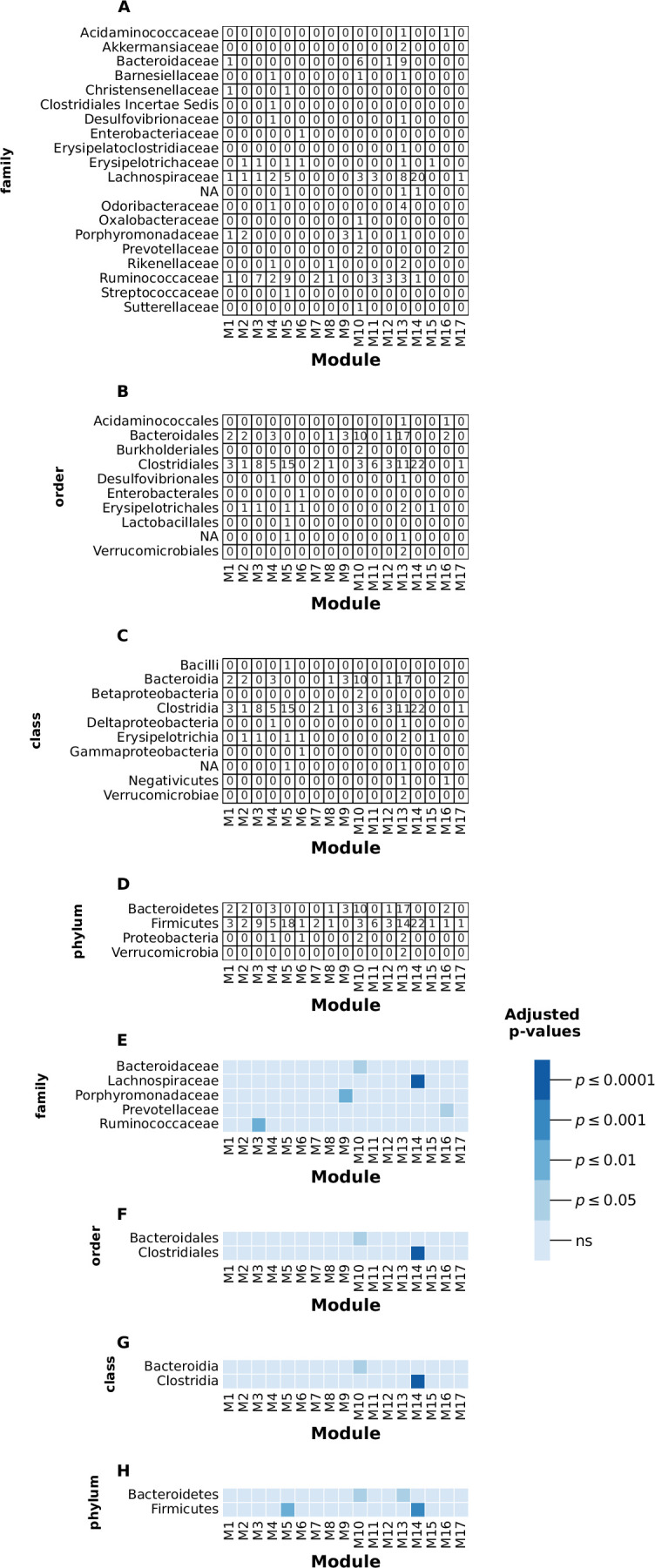
Taxonomic enrichment. Enrichment for the modules was performed at Phylum (**d**, **h**), Class (**c**, **g**), Order (**b**, **f**), and Family (**a**, **e**) levels using the hypergeometric test (one-sided), Methods §4.6.

To demonstrate the utility of the MDSINE2 software package for deriving biologically relevant information on ecosystem-scale microbiome dynamics from timeseries data, we performed an analysis of the healthy cohort data. MDSINE2 discovered 17 interaction modules (Fig. 5), ranging in size from 1 to 35 taxa, and connected through 56 interactions predicted with ‘decisive evidence’ (Bayes factor (BF) ≥ 100, Fig. 5c)^21^. This represents a 97% reduction in interaction parameters over MDSINE2^−M^ (2,179 edges predicted with ‘decisive evidence’; Extended Data Fig. 6), with nearly comparable forecasting performance as described above. As a basic measure of the biological relevance of modules, we evaluated the relatedness of taxa within modules and found that modules showed statistically significant enrichment for phylogenetic and taxonomic signals (Extended Data Figs. 7 and 8, and Supplementary Text 4.3.1).

To quantitatively evaluate the relative importance of each interaction module in the ecosystem, we performed a module-level keystoneness analysis^16^ (Fig. 5d and Methods section ‘Keystoneness’). In ecology, keystone taxa are defined as fundamental to the integrity of the ecological community^29^, and have been suggested as drivers of microbial community structure and function^30,31^. Here we extend the concept to groups of taxa (modules) and also generalize to a quantitative measure of ‘keystoneness’ with both positive and negative values. Positive keystone modules (‘promoters’) are those that, when removed, result in a reduction in the microbial abundances of the other members of the ecosystem; negative keystone modules (‘suppressors’) are those that, when removed, result in increases in abundances of the other members of the ecosystem. The magnitude of the keystoneness measure thus represents the degree of community-wide disruption (in terms of microbial abundance change, with the removal of the module).

**Extended Data Fig. 9:**
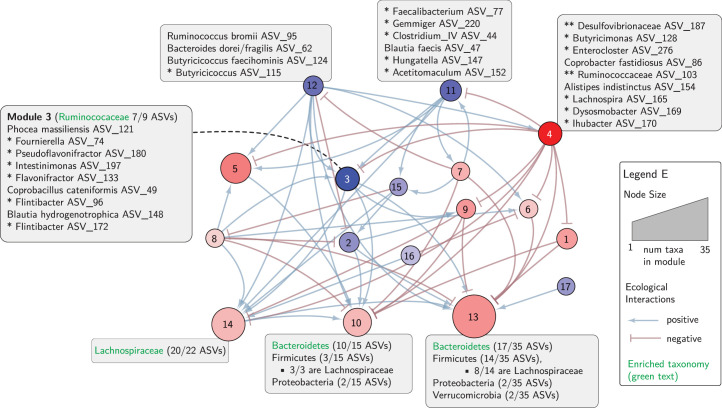
Annotated modules from Fig. 5c. Taxa from specific modules denoted by ASVs along with taxonomic breakdown at higher levels. Enriched taxonomies in green. Only to aid in discussion, not intended to be comprehensive.

For our cohort, the top positive and negative keystoneness modules were M3 and M4 respectively. Investigating their role through the ecological network, we see that all the outgoing edges of M3 are promoting, while all the outgoing edges of M4 are repressive, suggesting the different ecological roles that these modules play in the network. M3 is enriched for the family Ruminococcaceae. Promoting M3 are two other positive keystoneness modules M11 and M12, each containing taxa capable of degrading resistant starches (*Ruminococcus bromii* ASV95, *Gemmiger* ASV220) and others with butyrate production capabilities (*Faecalibacterium* ASV77, *Butyricicoccus faecihominis* ASV124, *Butyricicoccus* ASV115). Downstream modules being promoted by M3 of note are M10 (enriched for Bacteroidaceae), M13 (enriched for Bacteroidetes and the largest module in the network) and M14 (enriched for Lachnospiraceae). One explanation for this structure, consistent with known biology, is that the positive keystoneness modules are connected in a cross-feeding chain beginning with specialized starch-degrading taxa that ultimately support the more abundant generalist taxa (for example, Bacteroidaceae). In contrast to this specialist-to-generalist structure, the module with the highest negative keystoneness, M4, contains a diverse group of taxa and also suppresses multiple modules in the network, including M3 and M11, the top two positive keystoneness modules, as well as the primarily Gram-negative modules M10 and M13. An annotated module network is provided in Extended Data Fig. 9.

**Fig. 6:**
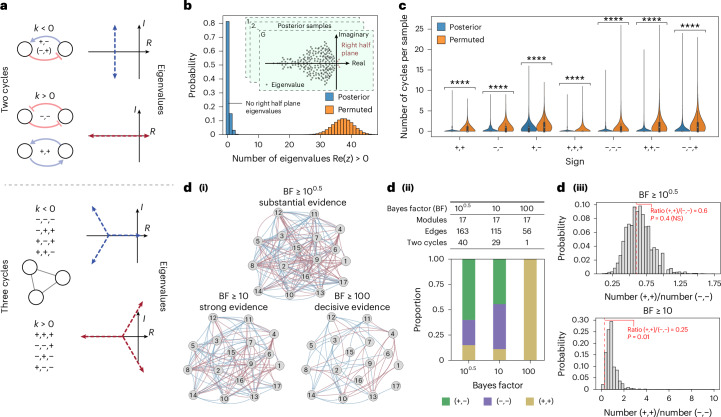
Analytical stability analysis of microbiomes demonstrates significantly more stable dynamics than expected by chance and reveals cycle feedback motifs. **a**, Two- and three-cycle motifs and their corresponding eigenvalue asymptotes for increased interaction strength. The cycle interaction strength is denoted by *k*. Axes are labelled with *I* and *R* to denote the imaginary and real axes, respectively. Only the two-cycle with negative feedback can remain stable for all feedback gains (top motif). **b**, As a measure of stability, we computed the number of right half plane eigenvalues for each posterior sample of our model trained on the healthy cohort (*n* = 100,000), and for the null model (*n* = 100,000 ‘permuted’ samples, one for each posterior sample). Our model had an 80% probability of having no unstable eigenvalues. **c**, Distribution of cycle motifs over posterior samples and permuted networks from **b**; all pairwise comparisons are based on BH-corrected two-sided Wilcoxon signed-rank test, ****P < 0.0001. In the violin plots, filled boxes denote interquartile region with a dot for the median; whiskers denote 1.5× the interquartile region. *P* values are provided in Supplementary Data. **d**, Analyses showing networks derived with three different levels of confidence for including edges (**i**), descriptive statistics for the networks (**ii**), and statistical test for significance of mutualism to competition ratio (MCR: (+,+)/(−,−)) (**iii**), which was significant for the network constructed from edges with ‘strong’ evidence *P* = 0.01. Significance was determined by permutation test comparing the topology in **d**(**i**) to 10,000 permutated networks. See Methods section ‘Network null model’ for network permutation details. Source data

To assess the overall robustness of our inferred ecosystem to external perturbations, we performed a formal stability analysis, which showed that the dynamics of the model inferred were 80% more likely to be stable than by chance (Fig. 6b and Supplementary Text 4.3.2). We next sought to identify features of the ecological network inferred by MDSINE2 that could explain this remarkable stability. Stability and control theory have established that the feedback cycle is the core topological feature driving stability^32^. Pairwise interactions, the simplest form of feedback cycles, have particular interpretations in ecology, and their contributions to stability are well characterized for linear and gLV dynamical systems^5^: mutualism (+,+) and competition (−,−) are destabilizing, and parasitism (+,−) is stabilizing. For length three cycles and higher, more complex ecological interactions arise, and any sign combination is potentially destabilizing (Fig. 6a)^33^. For all cycle lengths analysed, we found that MDSINE2’s inferred model of dynamics had a significantly lower number of cycles than expected by chance (Fig. 6c).

We next sought to understand the influence of uncertainty in the inferred network structure itself on stability estimates. To gain insight into this phenomenon, we evaluated networks at different levels of evidence for edges: ‘substantial’ [BF ≥ 10^0.5^], ‘strong’ [BF ≥ 10] and ‘decisive’ [BF ≥ 100] evidence (Fig. 6d(i) and Supplementary Text G). As the evidence threshold for an edge being included in the model was decreased, the number of edges in the network increased from 56 to 163 (Fig. 6d(ii)). As the number of edges increased, there was also an increase in the number of (two) cycles, as expected. Interestingly, for networks with more edges, the number of parasitism (+,−) cycles increased disproportionally among two cycles present, consistent with the property that stability becomes less likely the denser a network becomes (the more edges there are for a fixed number of nodes)^4,34,35^, unless the cycles in the network are only parasitism (Fig. 6a). Previous work has also examined the role of mutualism and competition cycles, hypothesizing that for healthy ecosystems, the mutualism to competition ratio (MCR: (+,+)/(−,−)) would be less than one^36^, and demonstrating this phenomenon on networks inferred on small microbial ecosystems^6^. Our results provide support for this hypothesis, showing that the mutualism to competition ratio was significantly lower than chance on networks with strong evidence for the existence of edges (Fig. 6d(iii)).

## Discussion

We have introduced MDSINE2, a computational method for accurately inferring interpretable dynamical systems models of the microbiome at scale, and demonstrated, using a new resource of densely sampled microbiome timeseries data from ‘humanized’ gnotobiotic mice and simulated data, that our approach outperforms other methods when forecasting microbiome dynamics or predicting ground-truth dynamics. Overall, MDSINE2 provides new tools for characterizing the dynamical systems behaviours of complex host–microbial ecosystems and holds promise for guiding rational design of interventions to stably alter human microbiomes for prophylactic or therapeutic purposes. MDSINE2 does have limitations and pitfalls, including modelling assumptions and data requirements, which we discuss in detail in Supplementary Text 5, along with some practical suggestions for applying our method and directions for future work.

Our case-study analysis additionally demonstrated MDSINE2’s ability to predict modules of bacteria with putative keystone roles in the healthy gut microbiome, elucidate possible cross-feeding hierarchies between modules and uncover interaction networks driving ecosystem stability. It will be essential to test these in silico predictions in future directed experiments. Testing of these types of hypothesis involving complex microbiomes remains an open and active area of research in the field, with many exciting approaches emerging. In general, isolation of bacterial strains in gut microbiomes and choosing appropriate in vitro growth conditions to recapitulate their in vivo ecological interactions remains challenging, although recent culturomics approaches hold promise^37,38^. Another promising approach are high-complexity but fully defined humanized mouse models, such as hCom^39^. These experimental systems allow for taxa subtraction and addition experiments, which could be used to probe ecological interactions and downstream functional consequences in complex microbiomes, analogous to knockout/gain-of-function-type experiments in cellular biology.

## Methods

This research complies with all relevant ethics regulations. Animal experiments were conducted under Brigham and Women’s Hospital (BWH) IACUC: 2016N000141, with human donor stool collected under BWH IRB: 2017P002420.

### MDSINE2 model

#### Overview

Our statistical model of microbial dynamics is a fully Bayesian model based on continuous-time stochastic generalized Lotka–Volterra dynamics:

1 $$\begin{array}{rcl}{\rm{d}}{{\boldsymbol{x}}}_{s,i}&=&{{\boldsymbol{x}}}_{s,i}(t)\left[\left(1+\mathop{\sum }\limits_{p=1}^{P}{{\boldsymbol{\gamma }}}_{p,{{\boldsymbol{c}}}_{i}}{{\boldsymbol{z}}}_{{p,{\bf{c}}}_{i}}^{\left(\gamma \right)}{h}_{p}\left(t\right)\right){{\boldsymbol{a}}}_{1,i}-{{\boldsymbol{a}}}_{2,i}{{\boldsymbol{x}}}_{s,i}(t)\right.\\ && +\left.\sum _{j:{{\boldsymbol{c}}}_{j}\ne {{\boldsymbol{c}}}_{i}}{{{\boldsymbol{b}}}_{{{\bf{c}}}_{i},{{\bf{c}}}_{j}}{\boldsymbol{z}}}_{{{\bf{c}}}_{i},{{\bf{c}}}_{j}}^{\left(b\right)}{{\boldsymbol{x}}}_{{sj}}(t)\vphantom{\mathop{\sum }\limits_{p=1}^{P}}\right]\,+{{\boldsymbol{x}}}_{s,i}(t){\rm{d}}{{\boldsymbol{w}}}_{s,i}.\end{array}$$

This formulation of stochastic behaviour models multiplicative random effects on microbial abundances, which could arise from a variety of phenomena, such as temporal host, environmental or dietary fluctuations that result in short-time-scale increases or decreases in abundance of each taxon.

The abundance of taxon *i* in timeseries *s* (for example, a biological replicate) is denoted as ${{\boldsymbol{x}}}_{{s,i}}$. MDSINE2 probabilistically assigns each taxon to an interaction module, where ${{\boldsymbol{c}}}_{i}$ denotes the module assignment for taxa *i*. The growth rate and self-interaction random variable for taxon *i* are denoted ${{\boldsymbol{a}}}_{1,i}$ and ${{\boldsymbol{a}}}_{2,i}$, respectively. The *P* external perturbations are accounted for by the random variables ${{\boldsymbol{\gamma }}}_{p,{{\boldsymbol{c}}}_{i}}$ that denote the effect of perturbation $p$ on taxa *i*’s growth rate; ${{\boldsymbol{z}}}_{{p,{\bf{c}}}_{i}}^{\left(\gamma \right)}$ is a corresponding random indicator variable that probabilistically selects whether the perturbation affects the interaction module. The function ${h}_{p}$ has a value of 1 during the timeperiod when the $p$ th perturbation is active and a value of 0 otherwise. The strength of the microbial interaction from taxon *j* to taxon *i* is denoted ${{\boldsymbol{b}}}_{{{\bf{c}}}_{i},{{\bf{c}}}_{j}}$, with ${{\boldsymbol{z}}}_{{{\bf{c}}}_{i},{{\bf{c}}}_{j}}^{\left(b\right)}$ being the corresponding random indicator variable for that microbial interaction. The stochastic variation in microbial abundances over time is captured by the variable ${{\boldsymbol{w}}}_{s,i}$, specifying geometric Brownian motion for the stochastic component (for example, a multiplicative stochastic process on the microbial abundance).

To support efficient inference, we used a first-order discretization (Supplementary Text) to yield the discrete-time latent trajectories:

2 $$\log \left({{\boldsymbol{x}}}_{s,i}\left(k+1\right)\right) \sim {\rm{Normal}}\left(\log \left({\mu }_{s,i}\left(k+1\right)\right),\,{{\Delta }}_{s,k}{{\boldsymbol{\sigma }}}_{w}^{{2}}\right),$$

where

$$\log \left({\mu }_{s,i}\left(k+1\right)\right)=\mathrm{log}\left({{\boldsymbol{x}}}_{s,i}\left(k\right)\right){\boldsymbol{+}}\,{{\Delta}}_{s,k}\left[{{\boldsymbol{a}}}_{1,i}\left({\boldsymbol{1}}{\boldsymbol{+}}\mathop{\sum }\limits_{p=1}^{P}{{\boldsymbol{\gamma }}}_{{{\boldsymbol{c}}}_{i},{\boldsymbol{p}}}{{\boldsymbol{z}}}_{{{\boldsymbol{c}}}_{i},{\boldsymbol{p}}}^{\left(\gamma \right)}{h}_{p}\left(k\right)\right)\right.$$$$\left.-{{\boldsymbol{a}}}_{2,i}{{\boldsymbol{x}}}_{s,i}\left(k\right)+\mathop{\sum}\nolimits_{j:\,{{\boldsymbol{c}}}_{j}\ne {{\boldsymbol{c}}}_{i}}{{\boldsymbol{b}}}_{{{\boldsymbol{c}}}_{{\boldsymbol{i}}}{,{\boldsymbol{c}}}_{j}}{{\boldsymbol{z}}}_{{{\boldsymbol{c}}}_{{\boldsymbol{i}}},{{\boldsymbol{c}}}_{j}}^{\left({\boldsymbol{b}}\right)}{{\boldsymbol{x}}}_{s,j}\left(k\right)\right]$$. Here, $${{\Delta }}_{s,k}={t}_{s,k+1}-{t}_{s,k}$$ is the difference between adjacent timepoints for the timeseries *s*. Below we give additional details on the model, including prior probability distributions on variables; for complete mathematical and algorithmic details, see Supplementary Text.

#### Interaction modules

We employed a Dirichlet Process (DP) prior^40^ to model interaction modules. The expected number of modules under this prior probability distribution is $\,\approx \alpha \log \frac{N+\alpha }{\alpha }$, where *N* is the number of taxa and $\alpha$ is the concentration parameter^41^. This property is desirable for scaling to large ecosystems, as the expected number of microbial interactions in our model scales as ${O(\log \left(N\right)}^{2})$ (as opposed to ${O(N}^{2})$ in the standard gLV model). We placed a diffuse Gamma prior on the concentration parameter as described in ref. ^42^. Our formulation allows us to marginalize out the interaction and perturbation parameters during inference, which greatly increases efficiency^40^. See Supplementary Text for complete details.

#### Interaction parameters and perturbation effects

To facilitate modularity and interpretability of inferred interaction networks, we assumed no intramodule interactions and model only intermodule interactions, ${{\boldsymbol{b}}}_{{{\boldsymbol{c}}}_{i},{{\boldsymbol{c}}}_{j}}$. We assumed that perturbations (for example, antibiotics or dietary changes) have module-specific effects, ${{\boldsymbol{\gamma }}}_{{{\boldsymbol{c}}}_{i}}$. Further, we modelled the presence/absence of module–module interactions and module-perturbation effects by using the binary indicator variables ${{\boldsymbol{z}}}^{({\boldsymbol{b}})}$ and ${{\boldsymbol{z}}}^{({\boldsymbol{\gamma }})}$, respectively. These binary indicators allowed the model to infer the structural edges that specify the underlying network topology between modules.

In addition, this formulation allowed for direct calculation of the statistical evidence for the presence of each interaction or perturbation effect using Bayes factors^21^. BFs are sometimes colloquially referred to as a Bayesian alternative to *P* values. Similar to *P* values, BFs quantitate evidence about hypotheses. Unlike *P* values, which quantitate evidence for a single hypothesis (the null hypothesis), BFs quantify evidence for and against two hypotheses or alternative models. In the context of the MDSINE2 model, we can thus use BFs to quantify the evidence of two competing models with an interaction (or perturbation) present or absent.

The BF is formally defined as the odds ratios of marginal likelihoods (the probability of data given a model, over all possible model parameters) of two competing models:

3 $${\rm{BF}}=\frac{{\rm{Probability}}\,{\rm{of}}\,{\rm{the}}\,{\rm{data}}\,{\rm{given}}\,{\rm{model}}\,1}{{\rm{Probability}}\,{\rm{of}}\,{\rm{the}}\,{\rm{data}}\,{\rm{given}}\,{\rm{model}}\,2}.$$

The numerator and denominator can then be rewritten using Bayes rule to yield:

4 $${\rm{BF}}=\frac{\frac{{\rm{Probability}}\,{\rm{of}}\,{\rm{model}}\,1\,{\rm{given}}\,{\rm{the}}\,{\rm{data}}}{{\rm{Probability}}\,{\rm{of}}\,{\rm{model}}\,1}}{\frac{{\rm{Probability}}\,{\rm{of}}\,{\rm{model}}\,2\,{\rm{given}}\,{\rm{the}}\,{\rm{data}}}{{\rm{Probability}}\,{\rm{of}}\,{\rm{model}}\,2}}.$$

The probability of a model given data is known as the marginal likelihood and the probability of a model is known as the prior. In our model, we are interested in the BF for the competing models of presence of an edge (model 1, *z* = 1) versus no edge (model 2, *z* = 0). Thus, the relevant BF is given by:

5 $${\rm{BF}}=\frac{{\rm{Marginal}}\,{\rm{likelihood}}({\boldsymbol{z}}=1)}{{\rm{Marginal}}\,{\rm{likelihood}}({\boldsymbol{z}}=0)}\times \frac{{\rm{Prior}}\,{\rm{probability}}\,({\boldsymbol{z}}=0)}{{\rm{Prior}}\,{\rm{pobability}}\,({\boldsymbol{z}}=1)}.$$

We used standard levels of evidence^21^ of ‘substantial’ (BF ≥ 10^0.5^), ‘strong’ (BF ≥ 10) and ‘decisive’ (BF ≥ 100) in this manuscript. See Supplementary Text Appendix G for a detailed derivation.

#### Measurement model

The observed data are sequencing counts ${{\boldsymbol{y}}}_{s,i}(k)$ of taxa and qPCR measurements ${{\boldsymbol{Q}}}_{s,{\rm{r}}}(k)$ of bacterial concentrations, where *j* indexes the qPCR measurement replicates. We modelled each measurement modality with its own distribution. Sequencing counts were modelled using a negative-binomial distribution^43^:

6 $${{\boldsymbol{y}}}_{s,i}\left(k\right)\,|\,{{\boldsymbol{x}}}_{s,i}\left(k\right),\,{r}_{s,k} \sim {\rm{NegBin}}\left(\varphi \left({{\boldsymbol{x}}}_{s,i}\left(k\right),{r}_{s,k}\right),\epsilon \left({{\boldsymbol{x}}}_{s,i}\left(k\right),\,{d}_{0},{d}_{1}\right)\right),$$

7 $$\varphi \left({{\boldsymbol{x}}}_{s,i}\left(k\right),{r}_{s,k}\right)={r}_{s,k}\frac{{{\boldsymbol{x}}}_{s,i}(k)}{\sum _{j}{{\boldsymbol{x}}}_{s,j}(k)},$$

8 $$\,\epsilon \left({{\boldsymbol{x}}}_{s,i}\left(k\right),\,{d}_{0},{d}_{1}\right)=\,\frac{{d}_{0}}{{{\boldsymbol{x}}}_{s,i}(k)/\sum _{j}{{\boldsymbol{x}}}_{s,j}(k)}+{d}_{1}\,.$$

Here, ${r}_{s,k}$ is the total number of reads for subject *s* at time ${t}_{s,k}$, and ${d}_{0}$ and ${d}_{1}$ parameterize the function $\epsilon (\cdot )$, which specifies the negative-binomial distribution dispersion parameter. We fit the parameters ${d}_{0}$ and ${d}_{1}$ using data from replicates (see below).

We modelled the qPCR measurements with a lognormal distribution:

9 $$\log \left({{\boldsymbol{Q}}}_{s,r}(k)\right) \sim {\rm{Normal}}\left(\log \left(\sum _{i}{{\boldsymbol{x}}}_{s,i}\left(k\right)\right),\,{\sigma }_{{{\boldsymbol{Q}}}_{s}(k)}^{2}\right).$$

Here, ${\sigma }_{{{\boldsymbol{Q}}}_{s}(k)}^{2}$ is the empirical variance of the set of qPCR measurement replicates for timeseries *s* at time ${t}_{s,k}$. For the ‘read-only’ variants of MDSINE2 (RO-MDSINE2, RO-MDSINE2^−M^) that were trained without qPCR measurements, the model was provided the following prior:

10 $$\log \left(\sum _{i}{{\boldsymbol{x}}}_{s,i}\left(k\right)\right)\, \sim {\rm{Normal}}\left(\log \left({10}^{10}\right),\,{0.1}^{2}\right)$$

for the total bacterial concentration. See Supplementary Text for complete mathematical details of the measurement model and inference procedure.

#### Priors and hyperparameters

Hyperparameters for all top-level prior probability distributions, except for the prior influencing the existence of module–module interactions, were set to be diffuse (uninformative) and thus favour the influence of the data on the posterior probability distribution over prior information. For the prior influencing the existence of module–module interactions, as in our previous work^16^, we used a strong prior to encourage sparsity for the analysis of the real data in the healthy cohort, that is, a prior expectation for no interactions present (Supplementary Text 1.4.1). When performing inference on the semi-synthetic data, we were interested in recovering the ground-truth network as accurately as possible and in that scenario, we used a non-informative prior for the module–module interaction indicators (Supplementary Text 1.4.1). To achieve diffuse priors for other variables, including those influencing taxa growth rates, self-interactions and module–module interaction magnitudes, mean values were set empirically from the data by fitting deterministic logistic growth curves to individual taxa, and variances were set by inflating the medians of empirically derived variances by 10,000×. For instance, this procedure calculated the hyperparameters for the prior on growth rates to be a mean microbial doubling time of ~0.7 days, with ~67% of values falling between 30 min and 3 days. A formal sensitivity analysis was performed for choices of settings for hyperparameters of priors influencing key variables in MDSINE2, including taxa growth rates, module–module interaction magnitudes and the existence of module–module interactions (Supplementary Text 3), showing insensitivity of cross-validated trajectory predictive performance to settings of hyperparameters across six orders of magnitude.

#### Software

MDSINE2 was implemented in Python 3.7 using the Numpy^44^, Scipy^45^, Numba^46^, Matplotlib^47^ and Seaborn^48^ packages. The software is publicly available under the Gnu General Public License v.3.0 (https://github.com/gerberlab/MDSINE2). The input to MDSINE2 consists of five tab-delimited files: (1) list of the sequence and taxonomic label for each taxa, (2) table of counts for each taxon in each sample, (3) table specifying the timepoints at which each sample was collected for each subject, (4) table of qPCR values for each sample and (5) table of perturbation names, start times, end times and associated subjects that received the perturbation. The software outputs inference results in two files: (1) a Python pickle file that contains the MDSINE2 inference objects and (2) an HDF5 file containing all the Markov chain Monte Carlo (MCMC) posterior samples. Once inference is complete, the software includes functionality to visualize and interpret the posterior samples, including visualizing trajectories, module networks (with a Cytoscape^49^ export option) and keystoneness, as well as generating text files with summaries of posterior distributions. See online software documentation for complete details. We also give demos of the functionalities in the tutorials. The tutorials (Google Colab) can be accessed from GitHub at https://github.com/gerberlab/MDSINE2_Paper.

#### Runtime analyses

**Extended Data Fig. 10:**
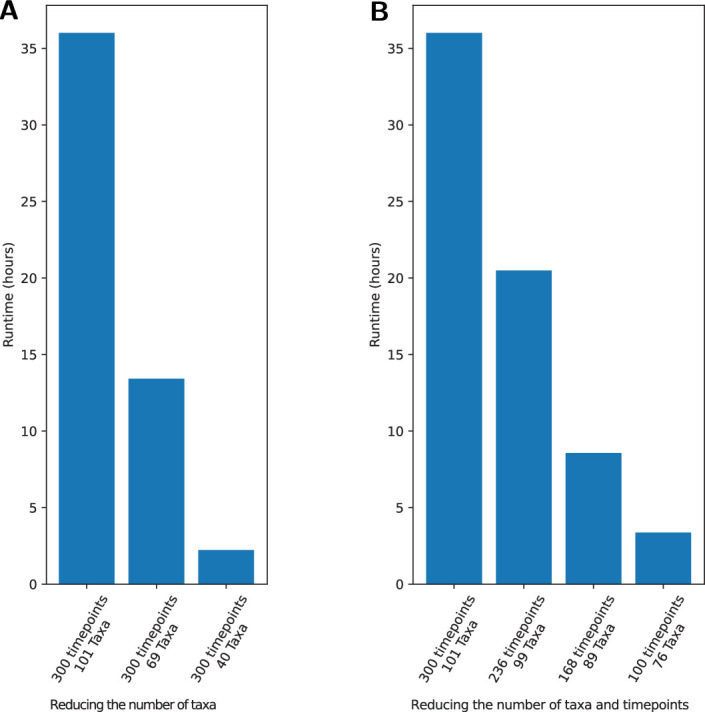
Runtime analysis. (**a**) Runtimes based on the semi-synthetic network from Fig. 5 with three different sets of taxa. (**b**) Runtimes based on the semi-synthetic network from Fig. 5 with varying numbers of timepoints and taxa.

All runtime analyses (Extended Data Fig. 10) were performed on a machine with an Intel i9-12900KF CPU with a performance core base frequency of 3.20 GHz and RAM of 128 GB. To assess how the number of taxa or timepoints changed the runtime, we varied both variables using our semi-synthetic data generation approach as described in the main text and below. Note that because our filtering criteria consider taxa to be present if their abundances are above a threshold for a set number of consecutive timepoints, reducing the number of timepoints also generally resulted in more taxa being filtered out. Overall, we observed more dramatic decreases in runtime with the number of taxa than timepoints (that is, ~1.8 h per taxon when reducing from 101 to 89 taxa versus ~0.25 h per timepoint when reducing from 300 to 236 timepoints). We hypothesize that this difference is due to our inference algorithm’s parallelization of some steps handling timeseries filtering, whereas taxa must be handled sequentially in our MCMC framework.

### Gnotobiotic experiments and microbiome data generation

#### Mouse experiments

Two cohorts of ~8–10-week-old male C57Bl/6 germ-free mice were used in the experiments (BWH IACUC: 2016N000141). All mice were derived from our germ-free colony at the Massachusetts Host-Microbiome Center (MHMC) at Brigham and Women’s Hospital. Mice were singly housed in Optmice cages within the MHMC. The mice were given a faecal microbiota transplant (FMT) from either a healthy human stool donor (four mice) or from a human donor with ulcerative colitis (five mice) from an ongoing study at Brigham and Women’s Hospital (IRB: 2017P002420). Following the study protocol, samples were flash frozen without cryoprotectants and stored at −80 °C. Material for FMTs was prepared by thawing the stool samples and homogenizing in 5 ml of pre-reduced 1× phosphate buffered saline with 0.05% cysteine inside an anaerobic chamber. Germ-free mice were then orally gavaged with 200 µl of FMT material per mouse. Post gavage, mice were equilibrated for 3 weeks before beginning a series of three perturbations: high-fat diet (HFD), vancomycin and gentamicin (in that order). Each perturbation lasted for 1 week, followed by a 1-week normalization period off perturbations. Aside from the HFD perturbation, mice were maintained on standard MHMC gnotobiotic mouse chow (Autoclavable Mouse Breeder Diet 5021; LabDiet). For the HFD perturbation, Research Diets D12492 (60 kcal% of fat) was used. For the vancomycin perturbation, drinking water was replaced with water containing vancomycin at a concentration of 100 μg ml^−1^ and 3% sucralose (filter sterilized). For the gentamicin perturbation, drinking water was replaced with water containing gentamicin at a concentration of 4 μg ml^−1^ and 3% sucralose (filter sterilized). In all situations, mice were allowed to eat and drink ad libitum. Mouse faecal pellets were collected in triplicate on the basis of the sample collection timeline detailed in Fig. 2. We also obtained additional samples to generate data for fitting the d0 and d1 parameters in our amplicon sequencing measurement noise model. For this purpose, a total of 9 faecal pellets (3 pellets on each of the 3 consecutive days 8, 9, 10) were collected from mouse 2. Each faecal pellet was divided into two parts. This resulted in 18 samples that were then processed through the entire sequencing pipeline, from DNA extraction through sequencing. To collect faecal pellets, each mouse was removed from the Optimice cage and placed inside an autoclaved Nalgene cup. After pellets were produced, mice were returned to their cages and samples were collected from the cup with autoclaved forceps. Samples were placed in cryovial tubes and snap frozen in liquid nitrogen immediately, then stored at −80 °C. At the end of experiments, mice were euthanized by an overdose of inhaled vapours of isoflurane administered in an anaesthesia chamber, followed by cervical dislocation. These procedures are in accordance with the recommendations of the Panel on Euthanasia of the American Veterinary Medical Association.

#### DNA extraction, 16S rRNA amplicon sequencing and qPCR

For DNA extraction, all samples were processed using the standard protocol^50^ at the MHMC, which used the Zymo Research ZymoBIOMICS DNA 96-well kit according to manufacturer instructions with the addition of bead beating for 20 min. Amplicon sequencing and qPCR were also performed using the standard MHMC protocol. Briefly, for amplicon sequencing, the v4 region of the 16S rRNA gene was PCR amplified using 515F and 806R primers^51^, 5’-[Illumina adaptor]-[unique bar code]-[sequencing primer pad]-[linker]-[primer]:

- (fwd primer): AATGATACGGCGACCACCGAGATCTACAC-NNNNNNNN-TATGGTAATT-GT-GTGCCAGCMGCCGCGGTAA
- (rev primer): CAAGCAGAAGACGGCATACGAGAT-NNNNNNNN-AGTCAGTCAG-CC-GGACTACHVGGGTWTCTAAT

Following PCR of the v4 region, 250-bp paired-end reads were generated on an Illumina MiSeq with the following custom primers with index primer, ATTAGAWACCCBDGTAGTCC-GG-CTGACTGACT:

- 5’-[sequencing primer pad]-[linker]-[primer] Read 1: TATGGTAATT-GT-GTGCCAGCMGCCGCGGTAA
- 5’-[primer]-[linker]-[sequencing primer pad] Read 2: AGTCAGTCAG-CC-GGACTACHVGGGTWTCTAAT

Total bacterial concentration estimation was performed with qPCR using universal 16S rRNA primers^50^:

- 1048F: GTG STG CAY GGY TGT CGT CA
- 1175R: ACG TCR TCC MCA CCT TCC TC

with a standard curve prepared from dilutions of *Bacteroides fragilis* (ATCC 51477). Samples were loaded into 384-well plates via the Eppendorf EP Motion liquid handler and then run on a QuantStudio 12K Flex Real-Time PCR System (ThermoFisher) using *Taq*Man Universal Master Mix II no UNG kit (ThermoFisher, 4440040), *Taq*Man Gene Expression Assay (ThermoFisher, 4331182), probe set dye (FAM, Quencher: NFQ-MGB) and reference dye (Rox for quantification; ThermoFisher, assay ID Pa04230899_s1), all according to manufacturer instructions.

### Bioinformatics

#### Generating ASV tables from amplicon reads

We generated an ASV read count table and assigned taxonomy using DADA2 v.1.16 according to the standard pipeline using pseudo-pooling^28^. Forward reads were trimmed to a length of 240 and reverse reads were trimmed to a length of 160. Our function calls for these core steps in the DADA2 pipeline were:


out <- filterAndTrim(fnFs, filtFs, fnRs, filtRs, truncLen=c(240,160), maxN=0, maxEE=c(2,2), truncQ=2, rm.phix=TRUE, compress=TRUE, multithread=TRUE)errF <- learnErrors(filtFs, multithread=TRUE, randomize=TRUE, nbases=1e8, pool = "pseudo")errR <- learnErrors(filtRs, multithread=TRUE, randomize=TRUE, nbases=1e8, pool = "pseudo")dadaFs <- dada(filtFs, err=errF, multithread=TRUE, pool = "pseudo")dadaRs <- dada(filtRs, err=errR, multithread=TRUE, pool = "pseudo")


To assign taxonomic labels to ASVs, we used DADA2-formatted reference databases RDP trainset 16 and Silva v.138. When using ‘assignTaxonomy’ in DADA2, we specified the maximum number of multiple species assignments to be 2. For species assignments, if one database returned a species assignment and the other did not, we labelled the ASV with the species from the database that returned the assignment. If both databases returned species assignments, but they were discordant, we set the assignment to the union of the returned assignments. If the total number of possible species assigned was greater than 4, then we did not set a species assignment.

#### Phylogenetic placement of sequences

We performed phylogenetic placement of consensus ASVs onto a reference tree constructed from 16S rRNA sequences of type strains tagged as ‘good’ quality, length between 1,200 bp and 1,600 bp in RDP (11.5)^52^. We performed multiple alignment of the sequences using the RDP’s web-hosted alignment tool with default parameters^53^. To facilitate a good multiple alignment, we filtered out sequences with insertions seen in $\le 3$ other sequences. A reference tree was constructed using FastTree^54^ v.2.1.7 SSE3 with the general-time-reversible maximum-likelihood option. For phylogenetic placement, the aligned reference sequences were first trimmed to positions 1,045–1,374 (corresponding to the region flanked by the 16S v4 primers) and a hidden Markov Model was learned using ‘hmmbuild’ in HMMER (v.3.1)^55^. ASV sequences were then aligned using ‘hmmalign’ with the ‘-mapali’ option. Finally, the aligned sequences were phylogenetically placed using ‘pplacer v.1.1.alpha19’ with default settings^56^.

#### Fold-change analysis

Fold-change analysis was performed using DESeq2 (v.1.3.2.0)^57^. All default options were used, with features only kept if there were at least 100 reads (summing across all the samples used in the analysis) using the following commands:


coldata$window < - factor(coldata$window)dds <- DESeqDataSetFromMatrix(countData = cts,colData = coldata, design = ~window)akeep <- rowSums(counts(dds)) >= 100dds <- dds[akeep,]dds < - DESeq(dds)


The scripts to perform this analysis can be found in GitHub at https://github.com/gerberlab/MDSINE2_Paper/tree/main/scripts/differential_abundance. The fold changes were calculated using the default Wald test in the software. The fold changes during the perturbations were calculated with respect to the ‘steady states’ achieved just before the perturbation was applied. The HFD fold change was calculated by comparing days (23, 23.5, 24, 25) to days (16, 18, 21, 21.5), the vancomycin fold change was calculated by comparing days (37, 37.5, 38, 39) to (32, 33, 35, 35.5), and the gentamicin perturbation fold change was calculated by comparing days (52, 52.5, 53, 54) to (46, 47, 50, 50.5).

### MDSINE2 analyses

#### Case-study model inference

Days 0 and 0.5 samples were excluded from inferences due to their very low overall bacterial concentrations. First, using replicate data, we performed a fit for the negative-binomial model resulting in ${d}_{0}=4.2\times {10}^{-8}$, ${d}_{1}=6.05\times {10}^{-2}$. Using these hyperparameters, inference was performed using 10 seeds configured to learn modules. For each seed, models were inferred using 10,000 MCMC iterations after 5,000 burn-in steps. Then, inference was performed on a single seed in fixed-cluster mode, where the consensus modules were derived using the concatenated outputs of the 10 seeds. To assess convergence of each Markov chain, we used the $\hat{R}$ statistic^58^ and confirmed values of $\hat{R}$ < 1.05 for the concentration parameter, a majority of the growth rates and the process variance, to indicate sufficient mixing. Full details on inference are given in the Supplementary Text.

#### Forecast benchmarking

We used implementations of the comparator methods provided in GitHub at https://github.com/tyjo/clv. Following ref. ^17^, we trained the models using elastic-net regression, and in addition, we trained gLV using ridge regression to provide comparisons to earlier work^14,16^. Predictive performance of methods was assessed using a hold-one-subject-out cross-validation procedure. Per fold, each method was provided data from all but one mouse in the cohort to infer model parameters. The inferred parameters were then used to forward simulate the trajectory of the held-out mouse, using the abundance at day 1 as the initial condition. For comparator methods, the Runge–Kutta ‘rk45’ procedure was used, as implemented in ref. ^17^. For MDSINE2, each posterior sample was used to deterministically forward simulate equation (2) with no process variance, and the median of the distribution of simulations was used as the final forecast. The methods use different approaches to handle zeros in data. To make results as comparable as possible, we used the following settings. For gLV-elastic-net and gLV-ridge, we set the minimum value for taxa to ${10}^{5}$ colony-forming units (c.f.u.s) g^−1^, which is consistent with the limit of detection in our experiments. For gLV-ra and LRA, which are relative abundance methods, we set the minimum to ${10}^{-6}$ and for CLV, we set the additive offset $\epsilon ={10}^{-6}$, consistent with the limit of detection for relative abundances in our experiments. These minimums were enforced both in data preprocessing and on the simulated trajectories, so that results remained comparable. For comparisons against methods that operate only on relative abundances (cLV, gLV-ra and LRA), we converted predictions of MDSINE2 or the gLV-based models to relative abundances. The following RMLSE metric (root-mean-squared logarithmic error) was used:

11 $${\rm{RMSLE}}\left({X}_{i,s},\,{\hat{X}}_{i,s}\right)=\sqrt{\frac{1}{K}{\sum _{k}\left({\log }_{10}{X}_{i,s}(k)-{\log }_{10}{\hat{X}}_{i,s}(k)\right)}^{2}}$$

where ${X}_{i,s}$ denotes the measurements for taxon *i* in the held-out mouse *s* and ${\hat{X}}_{i,s}$ are the respective forecast estimates. To compare the errors between MDSINE2 and other methods, we performed one-tailed Wilcoxon signed-rank testing. The paired datapoints used for the test are the RMSLEs associated with MDSINE2 and the RMSLEs associated with the comparator method for all the ASVs in the hold-out subjects.

#### Synthetic data

Benchmarking of models with synthetic data in Extended Data Fig. 4 followed the data generation procedure used to benchmark MDSINE in the original ref. ^16^ manuscript. Briefly, gLV dynamics were simulated for a community of 10 taxa with 10 biological replicates for a total of 30 days each. Nine taxa were simulated in the system at day 0 and the challenge taxon was introduced on day 10. Measurements were assumed to be daily, with qPCR measurements simulated from a lognormal distribution, and reads were simulated from a Dirichlet multinomial distribution.

#### Consensus module construction and fixed module inference

Consensus modules were constructed by performing agglomerative clustering on the co-clustering probability matrix where the number of clusters was the median number of modules over the posterior. See refs. ^22,23^ for additional details.

#### Semi-synthetic data and benchmarking

We generated a semi-synthetic dataset to mimic the scale and noise properties of real data. The data generation process was as follows:

1. Ground-truth dynamical system model: using 100,000 MDSINE2 posterior probability samples inferred from the healthy FMT cohort dataset of 141 taxa (10 different random seeds), we selected the dynamical system with the greatest forecasting capability (smallest cross-validated RMSE for predicted log abundances of taxa) and that predicted signal above the limit of detection (set to 10^5^ c.f.u.s g^−1^) in at least one mouse for at least one timepoint for each taxon (this criterion was included to at least allow for the possibility of predicting signal for all taxa in subsequent analyses). This dynamical system was then used as the ground-truth model.
2. Simulated ground-truth trajectories: lognormal distributions were fit for each of the 141 taxa, treating the 4 mice as biological replicates. Initial conditions were then sampled from these distributions 10 times, and trajectories were forward simulated from the ground-truth model defined in step 1 for each simulated mouse, resulting in 10 × 141 × 4 trajectories.
3. Simulated measurement noise: simulated observations were generated from the ground-truth trajectories using the MDSINE2 noise models for sequencing reads and qPCR data fit to technical replicates, which assumes the negative-binomial and lognormal distributions, respectively. For each trajectory, three different seeds were used to generate measurements.
4. Simulated temporal sampling: starting with the full set of timepoints at which faecal samples were collected in the real experiments, we downsampled trajectories to 3/4, 1/2 or 1/4 of the number of timepoints.
5. Filtering of simulated data: each simulated dataset was filtered using the same criteria as for real data, removing taxa not present at ≥0.01% relative abundance for seven consecutive timepoints in at least two mice.

For MDSINE2 models, interaction or perturbation coefficients were set to 0 if the associated indicator variable had a posterior probability ≤0.5, or set to the medians of the respective inferred posterior probability distributions otherwise. For other methods, which do not perform variable selection, the inferred coefficients were used directly. The AUC-ROC for determing whether two taxa were in the same module was determined by treating the co-clustering probability inferred by the model as a classifier. Specifically, for *n* taxa, we defined a binary $n\times n$ matrix where the $\left(i,j\right)$ th entry was 1 whenever taxon *i* was in the same module as taxon *j* in the ground-truth model. We then calculated an *n* × *n* matrix where the $\left(i,j\right)$ th entry was the posterior probability of *i* being in the same module as *j* for each inferred dynamical system, and compared it against the ground-truth matrix, ignoring the diagonal entries, resulting in an ${n}^{2}-n$ dimensional classifier to evaluate the AUC-ROC.

#### Taxonomic enrichment analysis

Using the consensus modules, we performed enrichment analysis at four taxonomic levels: family, order, class and phylum. The enrichment analysis was carried out using the hypergeometric test, followed by the Benjamini–Hochberg (BH) procedure for multiple hypothesis tests. The hypergeometric probability is defined as $${P}\left(X=k\right)=(\begin{array}{c}M\\ k\end{array})(\begin{array}{c}N-M\\ n-k\end{array})/(\begin{array}{c}N\\ n\end{array})$$. Here, *N* is the total number of ASVs used in the model, *M* is the total number of ASVs associated with a given taxonomic level, *n* is the size of the interaction module and *k* is the number of ASVs in the interaction module that is associated with the given taxonomic level.

#### Keystoneness

The keystoneness measure was computed by removing all the taxa for each module *m*, forward simulating trajectories (as described in ‘Benchmarking’) for the remaining taxa over 100 days and comparing the final state of these trajectories to the final state with all taxa present in the ecosystem. As in our perturbation experiments for stability analysis, final states were computed as the mean of values over the last 12 h in the final simulated day. To be precise, final state estimates ${x}^{(g)}$ (full system) and ${\widetilde{x}}_{m}^{\left(g\right)}$ (system with module removed) for each MCMC step *g*, which were used to compute the keystoneness measure, are given by:

12 $$k\left(m\right)=-\,{{\rm{mean}}}_{{\rm{g}}}\left[{{\rm{mean}}}_{i\notin m}\left({\log }_{10}\left({\widetilde{x}}_{{mi}}^{\left(g\right)}+\epsilon \right)-{\log }_{10}\left({x}_{i}^{\left(g\right)}+\epsilon \right)\right)\right],$$

where the subscript *i* denotes the taxon index. Just as in the simulation-based stability analysis, ${{\epsilon }}={10}^{5}$. Following this formulation, a positive keystoneness value indicates an overall decrease in the system on average (meaning *m* has a positive effect on other ASVs when present), while a negative keystoneness value indicates an increase (meaning *m* has a suppressive effect when present).

#### Stability

As a measure of stability, we computed the number of right half plane eigenvalues for the interaction matrix in each posterior sample. For a null model, we generated in-degree-preserving permutations of the interaction matrix (permutations are performed at the module level for each sample, see Methods section ‘Network null model’). The number of right half plane eigenvalues for the interaction matrix was then determined by counting the number of eigenvalues whose real part was greater than zero. For a theoretical discussion on the use of the eigenvalues of the interaction matrix for determining stability, see Supplementary Text 3.1.

#### Network null model

To provide a test of statistical significance for network topological features, we generated in-degree-preserving permutations of the interaction matrix, performed by permuting the off-diagonal elements in each row, to serve as null distributions of the network topological features. With this permutation, both the total number of edges in the network and the numbers of edges coming into each node remained the same, but the sources for the edges were uniformly randomly assigned with each permutation.

### Statistics and reproducibility

We followed our previous work for determining the number of mice in each cohort to produce sufficient information for dynamical systems inference^16^. Note that in the healthy cohort there were originally five mice; however, one of the mice (denoted subject or mouse 1 in the metadata tables) got out of the faecal collection container early in the longitudinal study (day 7) and had to be euthanized. We only analysed the healthy cohort with the four mice that had a complete timeseries (labelled subjects or mice 2–5). All data from mouse 1 were included in the raw sequences (https://www.ncbi.nlm.nih.gov/bioproject/PRJNA784519) and in the pre-processed data tables in GitHub (https://github.com/gerberlab/MDSINE2_Paper).

For differential abundance tests with DESeq2 (ref. ^57^), we used the two-sided Wald test. For all other testing, we either employed the non-parametric Wilcoxon signed-rank test (two-sided), hypergeometric test (one-sided) or bootstrapping via permutation. BH correction for multiple hypotheses was employed throughout the study. Data to reproduce components of figures where statistical tests were performed can be found in the Source Data files. All *P* values can be found in Supplementary Data. Scripts to reproduce figures in part or in whole can be found in GitHub at https://github.com/gerberlab/MDSINE2_Paper/tree/main/paper_figures.

### Reporting summary

Further information on research design is available in the Nature Portfolio Reporting Summary linked to this article.

## Supplementary information

Supplementary Information Supplementary Text.

Reporting Summary

Supplementary Data Raw and BH-corrected *P* values for all figures and module assignments for ASVs in the healthy cohort.

## Source data

Source Data Fig. 2 Raw data for plots with an associated statistical test.

Source Data Fig. 3 Raw data for plots with an associated statistical test.

Source Data Fig. 4 Raw data for plots with an associated statistical test.

Source Data Fig. 5 Raw data for plots with an associated statistical test.

Source Data Fig. 6 Raw data for plots with an associated statistical test.

Source Data Extended Data Fig. 1 Raw data for plots with an associated statistical test.

Source Data Extended Data Fig. 2 Raw data for plots with an associated statistical test.

Source Data Extended Data Fig. 3 Raw data for plots with an associated statistical test.

Source Data Extended Data Fig. 4 Raw data for plots with an associated statistical test.

Source Data Extended Data Fig. 5 Raw data for plots with an associated statistical test.

## Data Availability

All sequencing data for this study are available at https://www.ncbi.nlm.nih.gov/bioproject/PRJNA784519. Intermediate files for figure generation are available in Zenodo at 10.5281/zenodo.8208503 (ref. ^59^). Source data are provided with this paper.

## Code availability

First time users wanting to explore the model and the data should start by visiting the GitHub repository for this paper that reproduces figures and contains notebooks for exploring the model at https://github.com/gerberlab/MDSINE2_Paper (ref. ^60^). The core MDSINE2 package is also available in GitHub at https://github.com/gerberlab/MDSINE2 (ref. ^61^).
